# Menstrual Characteristics of Pubertal Girls: A Questionnaire-Based Study in Turkey

**DOI:** 10.4274/jcrpe.2026

**Published:** 2016-06-06

**Authors:** İhsan Esen, Baran Oğuz, Hepsen Mine Serin

**Affiliations:** 1 Fırat University Faculty of Medicine, Department of Pediatrics, Division of Pediatric Endocrinology, Elazığ, Turkey; 2 Fırat University Faculty of Medicine, Department of Pediatrics, Elazığ, Turkey

**Keywords:** adolescent, menstrual characteristics, dysmenorrhea

## Abstract

**Objective:** Clinicians should show an awareness on the menstrual characteristics of adolescent girls which may differ from adults in some aspects. To define menstrual cycle features among high school girls residing in a city center in southeastern Turkey.

**Methods:** A cross-sectional survey was conducted on 1256 girls attending a high school located in the city center of Elazığ, Turkey. Data from 879 girls (median age, 16.2 years; range, 13.6-19.2 years) who agreed to participate in the study and had started to menstruate were evaluated.

**Results:** Mean age at menarche was 12.7±1.3 years (range, 8.2-17.3 years). The mean cycle duration was 28.7±4.4 days, and the mean menstrual flow lasted 5.9±1.3 days. Severe, moderate, and mild dysmenorrhea was reported in 29%, 43%, and 28% of the girls, respectively, and 52% used analgesics for dysmenorrhea. A total of 34% of the girls defined their menstrual cycle as irregular, and 32% reported school absenteeism due to menstruation-associated complaints (pain and/or heavy bleeding). Menstrual bleeding affected attendance to classes and other school activities, daily work, social, family, and friend relationships, as well as sports/exercise activities in 43%, 49%, 58%, 48%, 44%, and 60% of the participants, respectively. In total, 30% of the responders had a problem with menstruation, and 12% and 17% of these stated that they consulted a primary care physician or specialist, respectively.

**Conclusion:** Dysmenorrhea was found to be common in adolescent Turkish girls and to affect daily life in approximately half of the girls.

## WHAT IS ALREADY KNOWN ON THIS TOPIC?

Menstrual-associated complaints are observed commonly in adolescents. In particular, pain accompanying the menstrual period is the most frequently reported complaint.

## WHAT THIS STUDY ADDS?

This study determined the menstrual characteristics of pubertal adolescent Turkish girls and the association between menstrual cycle features and interference with life activities.

## INTRODUCTION

Puberty is the period of human development during which secondary sexual characteristics appear, sexual maturation occurs, and reproductive capacity is attained. Ovulation and menstruation begin in girls during this period (1). Although this period usually passes without important problems, menstruation-associated complaints are observed commonly in adolescents. In particular, pain accompanying the menstrual period is the most frequently reported complaint (2,3,4,5,6,7,8). Irregular menstrual cycles are also reported frequently during the first years after menarche, possibly due to immaturation of the hypothalamic-pituitary-gonadal axis in the young adolescent (2,4). Physical, psychological, and emotional symptoms are also observed before and during menstruation in almost all adolescents (4,9,10,11). Mainly pain and also other symptoms occurring during menstruation affect daily life activities, can decrease school performance, and also increase the rate of school absenteeism (2,3,4,6,7,8,12).

In this study, we attempted to define the typical menstrual characteristics of adolescent girls residing in Elazığ city, Turkey and to investigate the effects of menstruation-associated complaints on life activities.

## METHODS

**Subjects and Setting**

The study was conducted in 2014 and consisted of a cross-sectional survey on girls attending six high schools randomly selected from different locations in the city center of Elazığ, Turkey. Questionnaires were distributed to 1256 girl students in grades 9-12 to be completed at home and 911 (72.5%) returned the completed questionnaire. Thirty-two students whose menstrual periods had not yet started were excluded from the analysis. Data from 879 pubescent girls who agreed to participate in the study and who had started to menstruate (median age, 16.2 years; range, 13.6-19.2 years) were evaluated.

The answers were transferred to an electronic database.

**Questionnaire Form**

The questionnaire form was prepared in Turkish and was based on a form used previously by Parker et al (4). The questionnaire was tried and assessed in 10 high school girls prior to the study. Based on the feedback, some complex statements were revised, and other questions were excluded. The final questionnaire comprised 82 questions divided into six sections. Students were asked to select from the options yes/no or true/false or to use a 1-10 scale for their answers. The questionnaire took about 20 min or less to complete. Menstrual pattern, duration and intensity of menstruation, school absenteeism due to menstruation, pain during the menstrual cycle, use of painkillers, physical-emotional symptoms during the menstrual cycle, and the effects of menstruation on various life activities were evaluated by the questionnaire. The menstrual period was considered regular if it occurred at intervals of 20-45 days. Pain during menstruation was categorized as severe (8-10 on a 0–10-point scale), moderate (4-7), or mild or no pain (0-3).

**Ethical Evaluation and Statistical Analysis**

The study protocol was approved by the Non-Interventional Studies Ethics Committee of Fırat University. Written informed consent was obtained from parents and the participating children.

The data were evaluated using the Statistical Package for the Social Sciences 18.0 program (SPSS Inc., Chicago, IL, USA). Incidence data are presented as percentages, and numerical data are expressed as means ± standard deviation. The chi-square test was used to compare categorical data, and the t-test was used to compare the numeric data. Statistical significance was accepted at p<0.05.

## RESULTS

The subjects had started their menstrual cycles 3.5±1.6 years previously. Mean age at menarche was 12.7±1.3 years (range, 8.2-17.3 years); 22 girls (2.5%) had experienced their first period before age 10 years, and 14 (1.6%) after age 15 years. The mean menstrual period duration was 28.7±4.4 days, and the mean length of menstruation was 5.9±1.3 days (range, 3-10 days) in 581 (66.1%) girls who defined their periods as regular. Approximately 62% (508/818) of the girls stated that their menstrual flow contained clots, whereas 5.2% (43/824) and 8.0% (66/824) stated that they experienced spotting before and in the middle of their menstrual period, respectively. Approximately 24% (213/879) of the girls defined their periods as irregular. Age at menarche and chronological age were comparable in girls with regular periods compared with those with irregular periods (p>0.05). Girls with irregular periods had a shorter duration of menstrual flow than did those with regular periods (3.2±1.6 vs. 3.5±1.5 days, respectively) (p<0.05). The girls who had reached menarche >2 years before the present study had significantly fewer irregular periods (48/506; 9.6%) compared to girls who had reached menarche ≤2 years before the study (165/373; 44.6%) (p<0.001).

The 10 most frequently reported symptoms are listed in Table 1.

**Pain During Menstruation (Dysmenorrhea)**

Approximately 92% of the responders had dysmenorrhea, and the pain was reported as severe, moderate, and mild or no pain in 28.8% (245/850), 43.3% (368/850), and 27.9% (237/850) of the subjects, respectively. Approximately 52% (412/793) of the girls reported that they used analgesics on their own during their menstrual cycle. Paracetamol was the commonest analgesic used (76%; 290/382), whereas 20% (77/382) and 3% (11/382) reported that they took oral and parenteral nonsteroidal anti-inflammatory drugs (NSAIDs). The data showed that 70% (319/457) of the girls who reported using an analgesic obtained a moderate to good (range, 4-10 on a 10-point scale) analgesic response from the drug. Only 1% (4/382) of the subjects had consulted a healthcare facility because of menstruation-related pain. Sixty-three (7.5%) girls reported that they never experienced pain during their period.

**Menstrual Cycle-Related Problems**

Approximately 12.0% (97/811), 16.7% (137/809), and 4.3% (38/807) of the subjects reported that they had consulted a primary-care physician, a specialist, or herbal drug dealer, respectively, for menstrual cycle-related problems. Analgesic tablets were recommended to 59 subjects for use during their menstrual cycle. Five subjects were prescribed oral contraceptive tablets because of a diagnosis of polycystic ovary syndrome.

**Effects of Menstruation on Life Activities**

Approximately 32% (261/822) of subjects were absent from school due to menstrual cycle-related reasons. Menstruation caused a 1 day absence for most girls (78.1%), but 2- (17.4%) and 3-day absences (4.5%) in 17.4% and 4.5% of the subjects, respectively. The causes for school absenteeism were pain in 88.2%, heavy bleeding in 4.9%, and nausea in 2.3% of the subjects. The effects of menstruation on seven groups of life activities in this group of subjects are shown in Table 2. Complaints related to menstruation and their effects on life activities are given in Table 3.

**Self-Perceptions of the Menstruation Cycle**

The perceptions of menstruating girls were evaluated in the true/false part of the questionnaire, which consisted of 30 statements. The 10 items most commonly considered to apply to the participant are shown in Table 4.

## DISCUSSION

Our results show that the menstrual characteristics of adolescent girls living in Elazığ, Turkey are similar to the typical characteristics reported in other studies (13). The duration of the menstrual cycle varies in adolescent girls, and their periods typically become regular as they age (14). Approximately half of the girls we surveyed who reached menarche <2 years previously reported irregular periods, whereas this number was very low in girls who had started menstruating >2 years ago. The extremes in menstrual cycle length are wider among adolescents as compared to adult women. However, a menstrual cycle beyond the 20-45-day range in young girls is not an expected finding and requires evaluation (13). No subject in our study reported periods more frequent than every 20 days, and a limited number of girls reported that they menstruated less frequently than every 45 days.

Previous studies show that pain usually accompanies menstruation in adolescents, with a reported pain incidence of 39-93% (2,3,4,5,6,7,8,15,16). In our study, the incidence of pain was at the upper limit and is the highest rate reported in Turkey. The frequency of analgesic use during menstruation was similar to rates reported previously (41-80%) (4,5,6). Girls in our cohort used paracetamol most often, which is consistent with previous findings, but more recent studies reveal a trend toward use of NSAIDs (4,6,17). A few subjects reported that they suffered from very severe pain during their periods that required a visit to a healthcare facility. These girls should be examined for possible pathologies, such as endometriosis. Parker et al (4) reported that 85% of subjects obtain a moderate-good analgesic response from the painkillers they use. We found a rate of 70%. However, both Parker et al (4) and our studies lacked sufficient data to determine whether the unsatisfactory analgesic responses were caused by too low doses, frequent drug use, or underlying serious pathologies, such as endometriosis.

Approximately one-third of the adolescents in our study reported severe pain during their menstrual cycle, which was similar to rates observed previously (4,6,7,9,15,18). The menstrual cycle-related school absenteeism rate of 32% observed here was also consistent with previous reports (2,3,4,6,7,8,9,12,15,18). Pain was reported to be the most common complaint causing school absenteeism (2,3,4,6,7,8). Similarly, our results showed that severe pain was the most common complaint causing school absenteeism during the menstrual cycle of Turkish adolescent girls. A few girls reported heavy menstrual bleeding as the cause of school absenteeism. We discovered that menstrual pain and symptoms substantially affected daily life activities of these adolescents. Similar results were reported previously in studies conducted in other countries (4,6,7,8).

Puberty is regulated by the hypothalamus through complex genetic mechanisms and is affected by ethnicity, nutritional status, and many other environmental factors. Age at menarche has become younger in developed countries since the mid-20th century, which has been associated with improved nutritional and economic status (19). This trend has stopped, probably because of stabilized socioeconomic status (20). The first study that investigated mean age at menarche in Turkish girls was conducted by Neyzi et al (21) in İstanbul in 1973, who found the mean age at menarche to be 12.4 years. Atay et al (22) conducted a study in İstanbul in 2009 and reported a mean age of menarche of 12.7 years. Our results show a mean age at menarche of 12.7 years, which supports the notion that the trend towards a younger menarche has stopped in Turkey, as reported by Atay et al (22).

To conclude, it may be said that the majority of the adolescent girls evaluated perceived their periods to be normal. However, mild to moderate dysmenorrhea with an impact on school attendance and social life was reported as a common occurrence. More effective and judicious use of painkillers can go a long way in ameliorating these problems. Also increasing awareness and educating adolescent girls and their parents regarding normal periods and related problems would have favorable effects such as a reduction in school absenteeism. Pediatricians interacting with adolescent girls should always discuss the menstrual periods, helping the girls and their families differentiate physiologic from pathologic states.

**Ethics**

Ethics Committee Approval: This present study was approved by the Ethics Committee of Fırat University in 2013, Informed Consent: It was taken.

Peer-review: External peer-reviewed.

## AUTHORSHIP CONTRIBUTIONS

Concept: İhsan Esen, Design: İhsan Esen, Baran Oğuz, Hepsen Mine Serin, Data Collection or Processing: İhsan Esen, Baran Oğuz, Hepsen Mine Serin, Analysis or Interpretation: İhsan Esen, Baran Oğuz, Hepsen Mine Serin, Literature Search: İhsan Esen, Writing: İhsan Esen.

Financial Disclosure: The authors declared that this study received no financial support.

## Figures and Tables

**Table 1:**
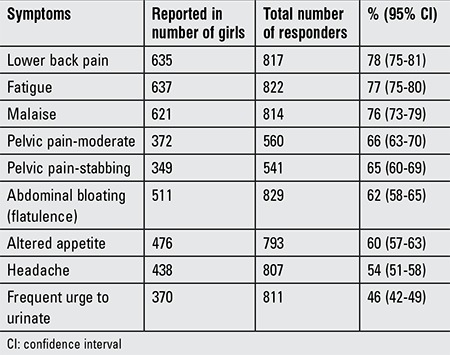
The 10 most common menstruation-related symptoms reported in the study group

**Table 2:**
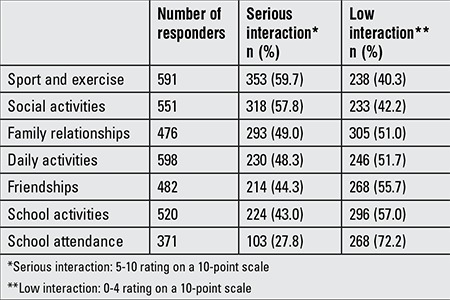
Effects of menstruation on life activities reported in the study group

**Table 3:**
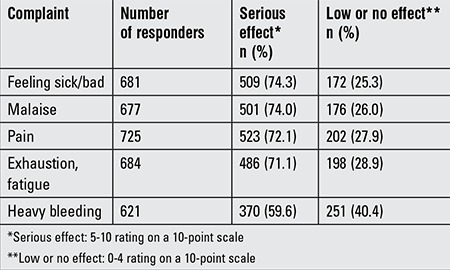
Complaints related to menstruation with serious effects on life activities reported in the study group

**Table 4:**
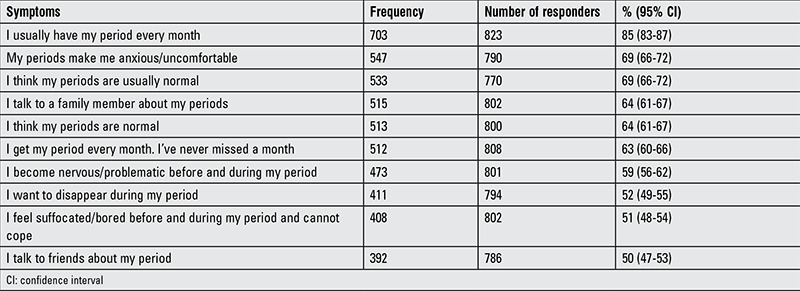
The 10 questionnaire items most commonly considered to apply to the study group
